# Tinnitus in patients with hearing loss due to mitochondrial DNA pathogenic variants

**DOI:** 10.1007/s00405-018-5028-y

**Published:** 2018-06-23

**Authors:** Urszula Lechowicz, Agnieszka Pollak, Danuta Raj-Koziak, Beata Dziendziel, Piotr Henryk Skarżyński, Henryk Skarżyński, Monika Ołdak

**Affiliations:** 1 Department of Genetics, World Hearing Center, Institute of Physiology and Pathology of Hearing, Mochnackiego 10, 02-042 Warsaw, Poland; 2 World Hearing Center, Institute of Physiology and Pathology of Hearing, Warsaw, Poland; 3 Department of Heart Failure and Cardiac Rehabilitation, Second Faculty, Medical University of Warsaw, Warsaw, Poland; 4 Institute of Sensory Organs, Nadarzyn, Poland; 5 Oto-Rhino-Laryngology Surgery Clinic, Institute of Physiology and Pathology of Hearing, Warsaw, Poland; 6 Department of Histology and Embryology, Medical University of Warsaw, Warsaw, Poland

**Keywords:** Tinnitus, Mitochondrial variants, Hearing loss, m.1555A>G, m.3243A>G, m.7511T>C

## Abstract

**Purpose:**

Tinnitus described as individual perception of phantom sound constitutes a significant medical problem and has become an essential subject of many studies conducted worldwide. In the study, we aimed to examine the prevalence of tinnitus among Polish hearing loss (HL) patients with identified mitochondrial DNA (mtDNA) variants.

**Methods:**

Among the selected group of unrelated HL patients with known mtDNA pathogenic variants, two questionnaires were conducted, i.e. Tinnitus Handicap Inventory translated into Polish (THI-POL) and Visual Analogue Scale (VAS) for measuring subjectively perceived tinnitus loudness, distress, annoyance and possibility of coping with this condition (VASs). Pathogenic mtDNA variants were detected with real-time PCR and sequencing of the whole mtDNA.

**Results:**

This is the first extensive tinnitus characterization using THI-POL and VASs questionnaires in HL patients due to mtDNA variants. We have established the prevalence of tinnitus among the studied group at 23.5%. We found that there are no statistically significant differences in the prevalence of tinnitus and its characteristic features between HL patients with known HL mtDNA variants and the general Polish population. In Polish HL patients with tinnitus, m.7511T>C was significantly more frequent than in patients without tinnitus. We observed that the prevalence of tinnitus is lower in Polish patients with m.1555A>G as compared to other available data.

**Conclusions:**

Our data suggest that the mtDNA variants causative of HL may affect tinnitus development but this effect seems to be ethnic-specific.

## Introduction

Tinnitus is a generally subjective perception of phantom sound, typically described as buzzing, ringing, hissing or beeping. Auditory hallucinations can be constant or intermittent with localization in one, both ears or within the head and may have different age of onset [1–4]. The etiology of tinnitus is broad and encompasses aging, hearing loss (HL), Meniere disease or environmental stressors such as noise, trauma or ototoxicity [5–7]. Studies in a group of 12,000 randomly selected Polish individuals have shown that tinnitus is present in approximately one in five adults (20.1%). About 1–3% of tinnitus patients suffer from debilitating tinnitus, which is usually associated with sleep disturbances, loss of productivity, psychiatric distress and poor quality of life [1, 8–10].

Studies regarding the genetic background of tinnitus, conducted in Swedish twin cohorts indicated that environmental factors may cause unilateral tinnitus, while genetic factors predispose to bilateral tinnitus, which may originate from a concomitant HL [11, 12]. In another study, 99 single nucleotide polymorphisms (SNPs) in ten genes involved in potassium recycling pathway in the inner ear were investigated in subjects exposed to occupational noise [13]. Statistically significant associations were obtained for genetic variants in the *KCNE1* and *SLC12A2* genes, however, the results have not been replicated in other populations. In the results of genome wide association study (GWAS) published by Gilles et al. [14], none of 4,000,000 SNPs reached the conventional threshold for genome-wide significance. However, using the genetic analysis of complex traits, it has been shown that variants which modify several metabolic pathways, such as oxidative stress, endoplasmic reticulum (ER) stress, and serotonin reception-mediated signaling, were significantly enriched in the tinnitus group [14].

Oxidative stress is identified as an important factor in the development of age-related hearing loss (ARHL), which is often accompanied by tinnitus. ARHL is combined with a decline of mitochondrial function due to increased reactive oxygen species (ROS) production [15–18]. Overproduction of ROS results in cytotoxic effects and oxidative stress may lead to HL and tinnitus [19].

In Polish HL patients, the most commonly studied pathogenic variants of the mitochondrial DNA are m.1555A>G in the *MT-RNR1* gene and m.3243A>G in *MT-TL1* [20, 21]. The pathogenic variant m.1555A>G causes an irreversible alteration in the 12S rRNA conformation, generating errors in protein synthesis [22] while m.3243A>G leads to deficient aminoacylation of tRNA^Leu(UUR)^, resulting in a reduced rate of mitochondrial protein synthesis and respiration defects [23]. *MT-TS1* gene encoding tRNASer^(UCN)^ is another HL-related hot spot in the mitochondrial genome with m.7511T>C located in its acceptor arm and disrupting its highly conserved structure, reducing by approximately 80% the level of tRNA synthesis and disturbing the mitochondrial protein translation [24].

Recently, it has been demonstrated that tinnitus patients have a considerably higher total oxidant status and oxidative stress index levels as compared to control subjects [25] and in a study conducted by Yano et al., 74.2% of patients with mtDNA variants causative of sensorineural hearing loss (SNHL) had tinnitus [26]. The data prompted us to investigate the prevalence of tinnitus among Polish HL patients with identified pathogenic mtDNA variants.

## Materials and methods

### Patients

For the study, we have included unrelated HL patients with variants in mtDNA [*n* = 41; 22 females (F) and 19 males (M)]. Medical records were retrieved from the genetic database of our Department of Genetics, Institute of Physiology and Pathology of Hearing (IFPS). Hearing status of the patients was determined based on pure tone audiometry at frequencies of 0.5, 1, 2, 4 and 8 kHz and classified according to the pure tone average (PTA) as mild (21–40 dB HL), moderate (41–70 dB HL), severe (71–90 dB HL) and profound (> 90 dB HL) [27]. In this group, major pre- and perinatal HL risk factors (i.e. severe prematurity, congenital rubella, mumps or cytomegalovirus infection, severe neonatal hyperbilirubinemia) as well as HL-causing DFNB1 locus pathogenic variants (tested according to the EMQN recommendations [28]) were excluded. Variants in mtDNA were detected by real-time PCR as part of a diagnostic workup of HL patients (m.1555A>G and m.3243A>G). Patients with previously identified m.7511T>C pathogenic variant were also included in the study [29].

A group of adult Polish individuals with tinnitus and normal hearing (*n* = 30) and patients with tinnitus and HL [genetic background of HL has not been tested (*n* = 67)], who underwent diagnostic tinnitus evaluation in Audiology and Phoniatrics Clinic of IFPS were used for statistical comparisons (Raj-Koziak et al. in preparation). The study was approved by the local Ethics Committee (IFPS:/KB/04/2012).

### Tinnitus Handicap Inventory translated into Polish (THI-POL), Visual Analogue Scale (VAS) of loudness, distress, annoyance and coping with tinnitus

Among the selected group of patients two questionnaires were conducted, i.e. Tinnitus Handicap Inventory translated into Polish (THI-POL) and Visual Analogue Scale (VAS) for measuring subjectively perceived tinnitus loudness (VAS-L), distress (VAS-D), annoyance (VAS-A) and possibility of coping with this condition (VAS-C) [30] (Raj-Koziak et al. in preparation). The THI-POL consists of 25 questions with the response choices “no” (0 points), “sometimes” (2 points) and “yes” (4 points). The score ranges from 0 to 100 (0–16 indicate slight, 18–36 mild, 38–56 moderate, 58–76 severe and 78–100 catastrophic tinnitus) [31]. VAS is a type of rating scale in which the subject ranks the health outcomes according to her or his preference and then places them on a line. VAS scores range on a scale from 0 to 100. The VAS scores were completed based on the patients’ answers determining the nuisance of each of these features over the last week. The loudness of tinnitus (VAS-L) was scored 0 whenever tinnitus was no audible and 100 if the patient has heard extremely loud sound. The VAS score for annoyance of tinnitus (VAS-A) was determined based on the patient’s subjective perception of annoyance (0 was scored for no annoyance and 100 for a maximal annoyance). The VAS score for the distress (VAS-D) was 0 if tinnitus was not distressing and 100 was given in case of an extremely distressing effect on life. The VAS score for coping with tinnitus (VAS-C) was 0 when the patient was coping completely and 100 if the patient could no longer cope with tinnitus (Raj-Koziak et al. in preparation). Additional questions such as the patient’s age at tinnitus onset, bilateral/unilateral tinnitus, usage of hearing aids (HAs) or cochlear implants (CI) were also recorded.

### Statistical analysis

Tinnitus prevalence and frequency of genetic variants between groups were compared by Fisher’s exact test or Chi-square statistics (with Yates’ correction if necessary). To compare VAS and THI-POL results between groups, *t* test was used. The data were considered statistically significant at *p* < 0.05.

## Results

From the patients included in the study, 17 individuals (17/41; 41.5%) participated in the survey (M = 7, F = 10). Mean HL degree and age of onset (AO) were 63.7 dB and 16.5 years old, respectively. In this group, four patients (4/17; 23.5%) confirmed tinnitus and 13 (13/17; 76.5%) denied its occurrence. The summary of clinical characteristics of tinnitus in our patients is given in Table 1. The mean age of tinnitus onset was 22.8 years. At the earliest, tinnitus occurred at the age of 7 in patient #1862 and as late as 30 in patients #1047 and #4912. All of the patients suffered from bilateral tinnitus and the majority of studied group (3/4; 75%) had constant tinnitus.

**Table 1:** Clinical characteristics of patients with HL mtDNA variants and tinnitus

Patient number	Sex	Tinnitus characteristics					HL characteristics			
		Tinnitus—LE, RE, bilateral	Age of onset (years)	Form of tinnitus	THI		Age of onset (years)	Mean HL (dB)	Usage of HA/CI	Mitochondrial variants
					Score	Severity				
118	F	Bilateral	24	Constant	36	Mild	37	84	LE HA, RE CI	m.7511T>C
1047	F	Bilateral	30	Intermittent	30	Mild	32	25	–	m.3243A>G
1862	M	Bilateral	7	Constant	10	Slight	15	30	–	m.1555A>G
4912	M	Bilateral	30	Constant	48	Moderate	7	74	Bilateral HA	m.7511T>C

*F* female, *M* male, *LE* left ear, *RE* right ear, *THI* Tinnitus Handicap Inventory, *HL* hearing loss, *HA* hearing aid, *CI* cochlear implant

**Fig. 1:**
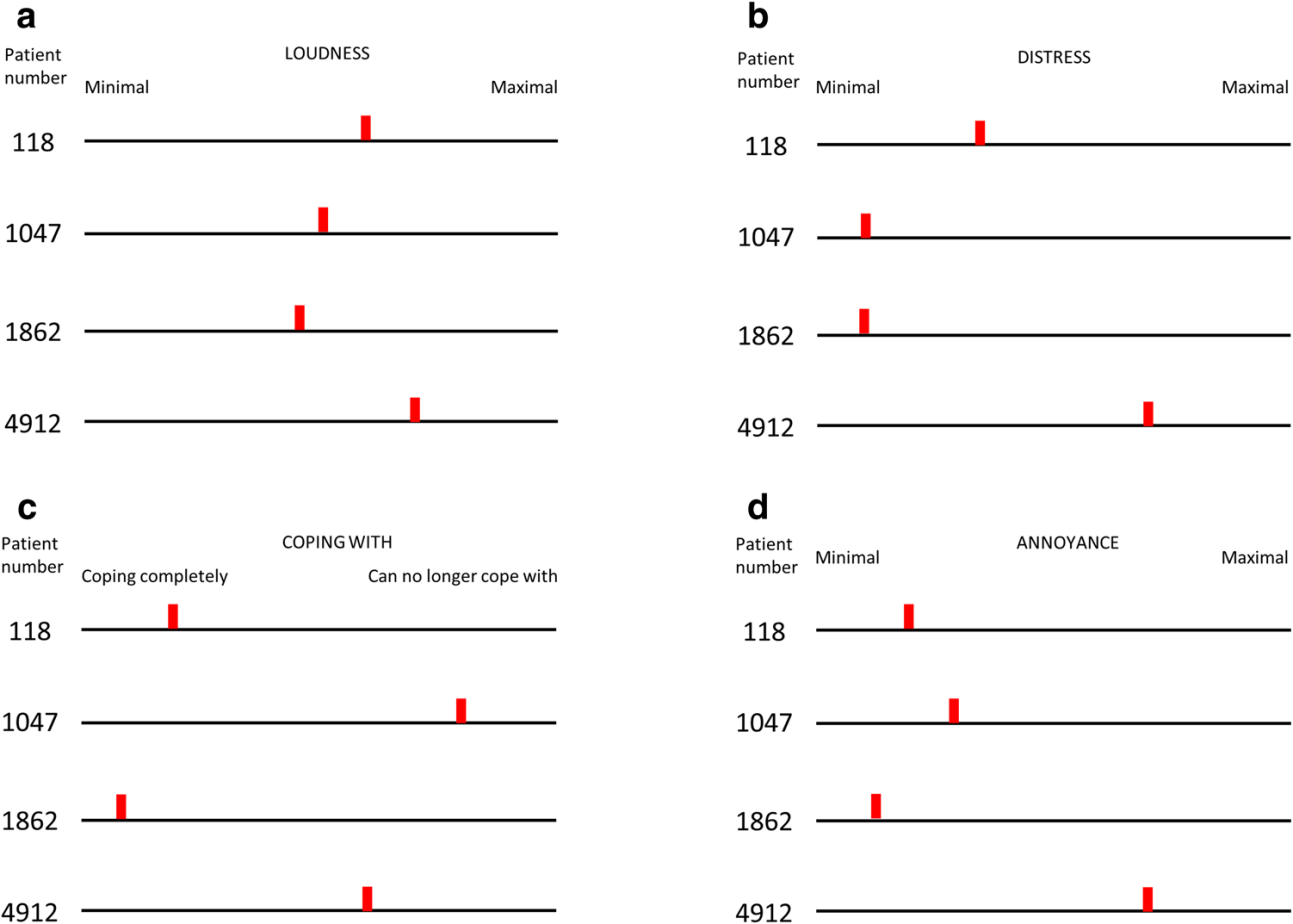
The results of VAS for loudness (**a**), distress (**b**), coping with (**c**) and annoyance (**d**) in four HL patients with mtDNA HL variants

The results of THI-POL ranged from 10 to 48 points, which indicates slight to moderate tinnitus. The results of VASs questionnaires are presented in Fig. 1. The loudness of tinnitus was assessed as medium by all patients. Almost all patients (except from patient #4912) determined their distress and annoyance caused by tinnitus as minimal. In case of coping, the results were scattered throughout the chart.

The age of HL onset in patients suffering from tinnitus was from 7 to 37 years old. In three subjects (patients #118, #1047, #1862) earlier occurrence of tinnitus than HL was observed. In these three patients the severity of tinnitus was slight or mild, while the severity of HL varied from mild (patient #1047) to severe HL (patient #118, #4912). In the fourth patient (#4912), HL occurred much earlier than tinnitus and both HL and tinnitus were relatively more severe in comparison with other patients. The m.7511T>C variant was identified in two patients (2/4), while m.1555A>G and m.3243A>G variants were found in one patient each (Table 1). Among patients who denied tinnitus there were seven individuals with m.3243A>G and six with the m.1555A>G pathogenic variant with the mean HL and HL AO reaching 65.9 dB and 14.5 years old, respectively.

We compared the frequency of each of the three mtDNA variants between patients with tinnitus and those who denied its occurrence. The difference was not statistically significant either for m.1555A>G or m.3243A>G but m.7511T>C was more prevalent in Polish patients with tinnitus (*p* = 0.0441). The prevalence of tinnitus between our HL patients with pathogenic mtDNA variants (4/17; 23.5%) did not differ from the general Polish population (2412/12,000; 20.1%) (*p* = 0.9603).

Next, we compared our results of each VASs parameter with the results obtained from adult Polish tinnitus patients with normal hearing (*n* = 30) and with HL (*n* = 67). There were no statistically significant differences between the compared groups for any of the four parameters (Table 2). Comparing our results of THI-POL survey with the group of 29 normal hearing patients with tinnitus (Raj-Koziak et al. manuscript in preparation) also did not reveal statistically significant differences (*p* = 0.63273).

**Table 2:** Mean scores for VASs parameters in three groups of tinnitus patients: (group 1) with HL and mtDNA pathogenic variants (*n* = 4), (group 2) patients with HL and unknown genetic background (*n* = 67), (group 3) patients with normal hearing (*n* = 30)

	Group 1		Group 2			Group 3		
	Mean	SD	Mean	SD	(Group 1 vs 2) *p* value	Mean	SD	(Group 1 vs. 3) *p* value
Loudness (VAS-L)	56.25	11.08	57.43	25.25	1	50.63	30.24	1
Distress (VAS-D)	31.25	28.39	59.64	28.19	0.39209	57.3	29.35	0.43608
Annoyance (VAS-A)	32.5	26.3	51.06	30.93	0.54182	47.2	30.84	0.54404
Coping with (VAS-C)	57.5	33.04	40.70	29.39	0.29403	48.17	34.6	0.44737

*SD* standard deviation

## Discussion

Here, we present the first comprehensive tinnitus characteristics in HL patients with mtDNA pathogenic variants that was conducted with THI-POL and VASs questionnaires, validated and recognized tools for tinnitus assessment. Tinnitus may arise as a consequence of cochlear hair cells injury or damage of the auditory pathway [6, 32, 33] but the molecular mechanism leading to tinnitus development is largely unknown. Changes in the structure of mtDNA due to occurrence of pathogenic variants may lead to dysfunction of mitochondria [34–36]. The dysfunctional organelles may in turn cause oxidative stress, which was claimed to play a role in the development of tinnitus accompanying sensorineural HL [37, 38]. Taking this into account, we have decided to investigate tinnitus in patients with HL due to mtDNA pathogenic variants.

Unexpectedly, in this group of patients, we did not find statistically significant differences in the prevalence of tinnitus as compared to the corresponding general population. Tinnitus characteristics in the studied group also did not significantly differ either from patients with tinnitus without HL or from patients with HL and tinnitus. The results suggested that mtDNA pathogenic variants leading to HL are not associated with tinnitus development, at least in our study group. The observation was reinforced by the data of Shargorodsky et al. who found a significant associations between ethnicity and tinnitus in individuals without hearing impairment and proposed that the mechanism of tinnitus development is independent of hearing impairment [8].

To the best of our knowledge, there is only one study on tinnitus and mtDNA HL variants [26]. It is confined to the Japanese population and there has been no reports on the involvement of HL mtDNA variants in the development of tinnitus in HL patients of other ethnicities. Analyzing the results we found that the prevalence of tinnitus (4/17; 23.5%) in our HL patients was much lower than in the cohort studied by Yano et al. [26], who identified tinnitus in 23 out of 31 HL patients (74.2%) with confirmed pathogenic mtDNA variants (*p* < 0.002). Interestingly, the prevalence of tinnitus in the Japanese patients (74.2%) was more than six times higher than that of the general Japanese population (11.9%; 1710/14,423; *p* < 0.0001) [39].

The association between m.7511T>C and tinnitus that was found in our study group, was not observed for the Japanese patients. For the remaining two variants, m.3242A>G and m.1555A>G, neither in Polish nor in Japanese patients, a statistically significant difference was found.

Analyzing the prevalence of tinnitus in patients with a particular mtDNA pathogenic variant, we have detected a statistically significant difference only for patients with m.1555A>G. The prevalence of tinnitus was almost six times higher in Japanese patients with m.1555A>G (13/16) as compared to our patient group with m.1555A>G (1/7) (*p* = 0.0049). For the remaining two mtDNA variants no statistically significant differences were found.

This revealed that in the Japanese study the incidence of tinnitus was significantly higher in comparison to our group. Considering the high prevalence of tinnitus in the Japanese HL patients and the possible differences in the prevalence of tinnitus among various populations [8], we expected a high prevalence of tinnitus in the general Japanese population and were surprised to find that it was even lower than in the general Polish population. A more than three times higher tinnitus prevalence in Japanese HL patients as compared to our study group indicated that HL-causing mtDNA variants are involved in tinnitus development in an ethnic-specific manner.

Among the HL-causative mtDNA variants m.1555A>G deserves a particular attention. Restricting the analyzed groups to patients with a single mtDNA variant we found that tinnitus was more prevalent in Japanese than in Polish patients with m.1555A>G, which indicates that the pathogenic variant plays an important role in tinnitus development in this population probably together with an yet unidentified predisposing factor(s). The results may also suggest that Polish patients carrying the m.1555A>G are not exposed to this risk factor or have other factors protecting them from tinnitus development. It is also worth to further investigate the role concerning m.7511T>C involvement in tinnitus development. There are only nine families reported worldwide with m.7511T>C pathogenic variant and some of the patients were reported to have tinnitus [29].

Our study underlines the ethnic-specific role of HL mtDNA variants in tinnitus development. The patient groups analyzed here differ in respect to race and geographic origin, which may be directly translated into differences in their genetic background and interaction of DNA with different environmental factors. Further studies involving larger patient groups are needed and since m.7511T>C and m.1555A>G has come to the forefront of potential tinnitus-associated mtDNA variants, broader research including these variants should be conducted in the future.
